# Unveiling the comparative efficacy and tolerability of comprehensive treatments for migraine: A protocol of systematic review and Bayesian network meta-analysis

**DOI:** 10.1097/MD.0000000000024083

**Published:** 2021-01-29

**Authors:** Boru Jin, Huayan Liu, Lei Qiao

**Affiliations:** a Department of Neurology, First Affiliated Hospital, China Medical University; b Department of General Surgery, Shengjing Hospital of China Medical University, Shenyang, People's Republic of China.

**Keywords:** Bayesian network meta-analysis, migraine, non-pharmacological treatments, pharmacological treatments, systematic review

## Abstract

**Background:**

Migraine is a chronic paroxysmal incapacitating neurological disorder, which endangers the health of human worldwide ranking as the third most prevalent medical condition. There are no comprehensive estimates of treatments for migraine. We will conduct this systematic review and Bayesian network meta-analysis (NMA) to synthesis quantitative and comparative evidence on the efficacy and tolerability of all the known pharmacological and non-pharmacological interventions for migraine.

**Method:**

We will perform the systematic electronic search of the literature utilizing MEDLINE, Embase, Cochrane Central Register of Controlled Trials (CENTRAL), Cumulative Index to Nursing & Allied Health (CINAHL), and PsycINFO. We will only include randomized controlled trials (RCTs) of high quality which appraise the efficacy or safety of any potential pharmacological or non-pharmacological interventions in the treatment of patients with migraine. The traditional pairwise meta-analyses will be performed to anticipate the heterogeneities and publication bias and the NMA will be conducted within a Bayesian hierarchical model framework to obtain estimates for all valuable treatments for migraine. The entire heterogeneity will be quantified by *Q* statistic and *I*^2^ index. Other analyses included sensitivity analyses, meta-regression, and subgroup analyses will also be conducted. The whole process will be conducted using in R-3.6.0 software.

**Results:**

This study will obtain the efficacy and tolerability of all potential treatments for migraine, aiming at providing consolidated evidence to help make the best choice of interventions. The results will be published in a peer-reviewed journal.

**Discussion:**

This Bayesian network meta-analysis may be the first attempt to quantitatively synthesize the efficacy and tolerability of all potential treatments for migraine. And this method can ensure us to fully utilize both the direct and indirect evidence as well as gain the comparative estimates displayed in the derived hierarchies. Besides, we have registered this protocol on the international prospective register of systematic review (PROSPERO) (CRD42020157278).

## Abstract 2

Supplemental Digital Content is available in the text

## 1 Introduction

### 1.1 Rationale

Migraine is a chronic paroxysmal incapacitating neurological disorder and manifested by attacks of moderate or severe headaches and reversible neurological and systemic symptoms such as photophobia, phonophobia, cutaneous allodynia, and gastrointestinal symptoms.^[1]^ Migraine seriously endangers the health of human worldwide that ranks as the third most prevalent medical conditions and the second most disabling neurological disorders.^[2,3]^ Migraine tortures 12% of the total population and mainly attacks the healthy young and middle-aged people, the data of which could even been underestimated globally.^[4,5]^ Women are 3 times more likely than men to suffer from migraine with a morbidity of 18%, and also may bear longer duration of headaches, more accompanying symptoms, severer related disability, and a higher burden of comorbidity, all of which would worsen with growing older.^[5,6]^ The rising morbidity of school-aged children and adolescents is 10% and that of high school-aged children has reached up to nearly 30%, which heavily damages their functional ability and quality of life and calls for our attentions urgently.^[7,8]^ Moreover, it is estimated that in the United States the unadjusted total health care expenditures on migraine is $56.31 billion per year, and thus the vast cost of migraine has pushed a huge burden on global economy.^[9,10]^ However, there are still no comprehensive estimates of treatments for migraine no matter the pharmacological or the non-pharmacological, let alone the quantitative comparative efficacy and safety.

The pharmacological medication has been the first-line approach treating migraine, and patients prefer to take them for prevention of attacks or acute attacks usually because of its convenience and efficacy rather than official guidelines.^[11]^ While due to the chronic course of migraine, patients always suffer from the risk of incurring in drug abuse, medication overuse headache as well as large amount of side effects.^[12]^ Therefore, taking an overall consideration of both the efficacy and the safety is significantly important. Although previous studies have provided some recommendations on the effective class of medications, they didn’t reach a consensus and all of them were based on the small number of clinical trials with biases.^[13,14]^ Besides, all the previous reviews only focus on limited fields of pharmacological treatments such as parenteral drugs,^[15]^ Chinese herbal medicine,^[16,17]^steroids,^[18–20]^or other specific medications,^[21–23]^ which result in numerous and sophisticated assessments without concluding a comprehensive guideline of pharmacotherapeutics for migraine.

Since the chronicity of migraine, the adverse events and tolerance of medication have perplexed patients a lot, which gives rise to the development of complementary and integrative medicine.^[24]^ Hence, more and more patients and clinicians turn to seek help from some burgeoning therapies including nutrition and diet alteration, movement practices, psychotherapeutics, manual therapy, acupuncture, mind-body strategies, magnetic stimulation, sleep intervention, Yoga, Tai Chi, spinal manipulation, massage, etc.^[24–27]^ Surgical strategies also play an essential role in treating refractory cases, for example, peripheral nerve decompression surgery could effectually reduce frequency and intensity of migraine.^[28]^ Generally, the comparative efficacy and tolerability of all the treatments for migraine no matter the pharmacological or the non-pharmacological have never been unveiled.

### 1.2 Our objective

We will conduct this systematic review and Bayesian network meta-analysis (NMA) as the first attempt aiming at answering the following questions. Among all available treatments of migraine, which are significantly statically effective for abortive or/and preventative medication, and are they safe? Do pharmacotherapeutics really benefit more than the non-pharmacotherapeutics as we used to thought? Which field or specific one of these treatments should be recommended first considering both efficacy and safety? Furthermore, this study would provide the comparative hierarchies of efficacy and safety of all the available treatments of migraine.

## 2 Method

Our systematic review and NMAs will be done across all types of interventions in order to derive comprehensive estimates of efficacy and safety on all potential therapies for migraine. We will perform this systematic review and NMA in conformity to Preferred Reporting Items for Systematic Review and Meta-Analysis Protocols (PRISMA-P).^[29]^ And the final completed study would strictly followed PRISMA-NMA extension statement.^[30]^ We have registered this protocol on the international prospective register of systematic review (PROSPERO) (CRD42020157278).

### 2.1 Criteria for included studies

#### 2.1.1 Participants and settings

The participants included in this NMA are all diagnosed as migraine according to the recognized criteria: International Classification of Headache Disorders (ICHD) for migraine headaches.^[31]^ We will exclude the studies and trials which recruit participants with “mixed” or “combination” migraine and other types of headache such as tension-type headache. There are no restrictions of sex, age, ethnicity, nationality, or duration of disease.

#### 2.1.2 Interventions

**Figure 1:**
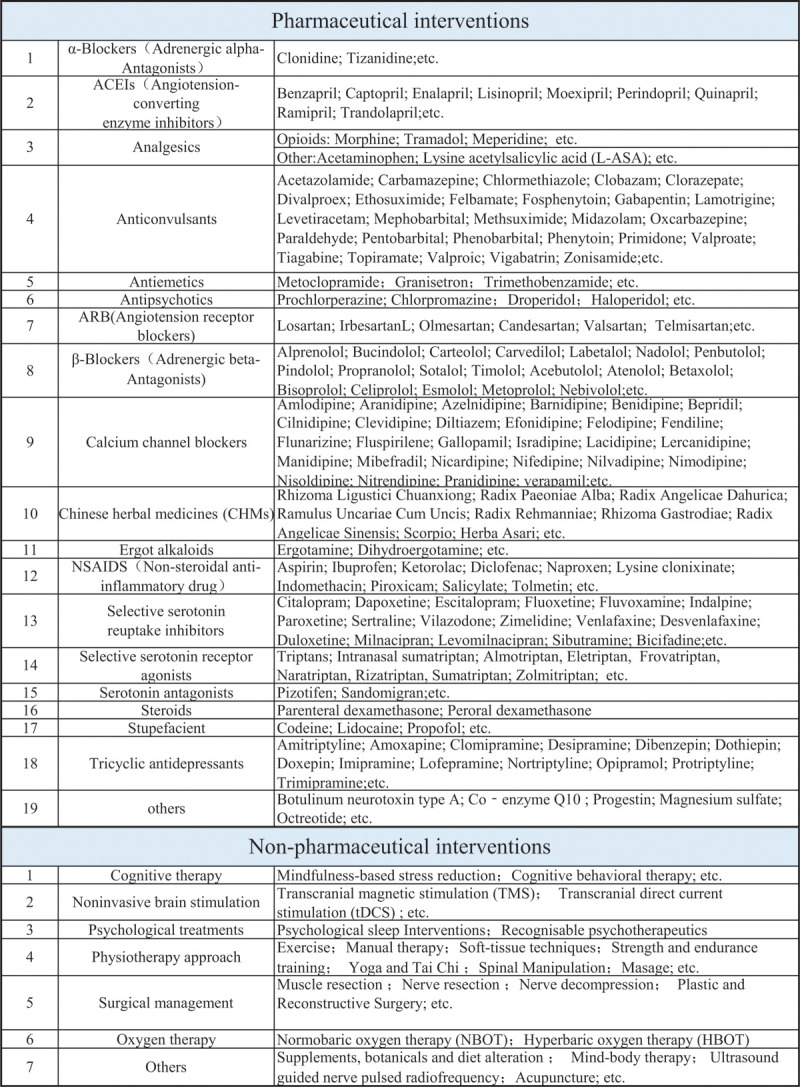
Searching key words of pharmacological and non-pharmacological therapies.

All the available treatments both pharmacological or non-pharmacological strategies for migraine will be considered in this NMA being accessing efficacy and safety. Given that the therapies for migraine is complicated and some are even used in “off-label” fashion, we will not restrict the interventions to only include experienced prescription or some old guidelines recommended in order to exploit all the potential treatments for migraine. Some fields of pharmacological drugs will be carefully retrieved such as adrenergic α-antagonists, angiotension-converting enzyme inhibitors, analgesics, anticonvulsants, antiemetics, antipsychotics, angiotension receptor blockers, adrenergic β-antagonists, calcium channel blockers, Chinese herbal medicines, ergot alkaloids, non-steroidal anti-inflammatory drugs, selective serotonin reuptake inhibitors, selective serotonin receptor agonists, serotonin antagonists, steroids, stupefacient, tricyclic antidepressants, etc. Non-pharmacological therapies will also be fully explored including cognitive therapy, noninvasive brain stimulation, psychological treatments, physiotherapy approaches, surgical management, oxygen therapy, etc. More specific potential pharmacological and non-pharmacological interventions are outlined in Fig. 1

#### 2.1.3 Comparators

Eligible comparator groups within this NMA include usual care, placebo, or another included pharmacological or nonpharmacological treatments for migraine.

#### 2.1.4 Outcome

After a literature review of previous reviews, considering both the power and the practicability of the scales or index, our outcomes will be carefully selected. Our primary outcomes are as follow. The number of participants with pain-free response at 2 hours (complete resolution of headache pain). The number of participants with headache pain reduction from moderate/severe to mild or none at 2 hours. The number of participants with sustained relief for 24 hours. Our secondary outcomes of efficacy included migraine disability assessment (MIDAS) questionnaire, Headache impact test (HIT-6), mindfulness attention awareness scale (MAAS), 36-item short form survey (SF-36), headache frequency per month, headache severity, and headache duration.

Our primary outcomes of safety include the total adverse events, restlessness, drowsiness, nausea, vomiting. Other side effects such as tingling, numbness, swelling and any other adverse events would also be analyzed as the secondary outcomes.

#### 2.1.5 Study designs

Only randomized controlled trials (RCTs) in English will be included in our systematic review and NMA in order to provide high quality of evidence for the comparative efficacy and safety of migraine strategies. Therefore, qualitative studies, observational studies, meta-analyses, case reports, case series, ecological studies, and policy papers will be excluded. Some the non-randomized trials and publications which were not peer-reviewed (such as conference proceedings, letters, and comments) would also be ruled out.

### 2.2 Information sources and search strategy

We will perform the systematic electronic search of the English-language literature utilizing databases of MEDLINE, Embase, Cochrane Central Register of Controlled Trials (CENTRAL), Cumulative Index to Nursing & Allied Health (CINAHL), and PsycINFO. Searching strategy is designed characteristically for each database, which is a combination of free text, Medical Subject Heading, EMTREE terms, etc (Supplementary 1, http://links.lww.com/MD/F541). The search dates will be from the inception of the respective library to November 31, 2020, and another search of each database and registration platform would be retrieve again before completing this NMA for fear of leaving out any newly published works. The unpublished studies will be retrieved via conference proceedings, clinical trial registries, and author contact. The reference lists of included studies and related reviews will be scanned carefully only to identify potential studies for inclusion in our NMA.

### 2.3 Data collection and analysis

#### 2.3.1 Study selection

**Figure 2:**
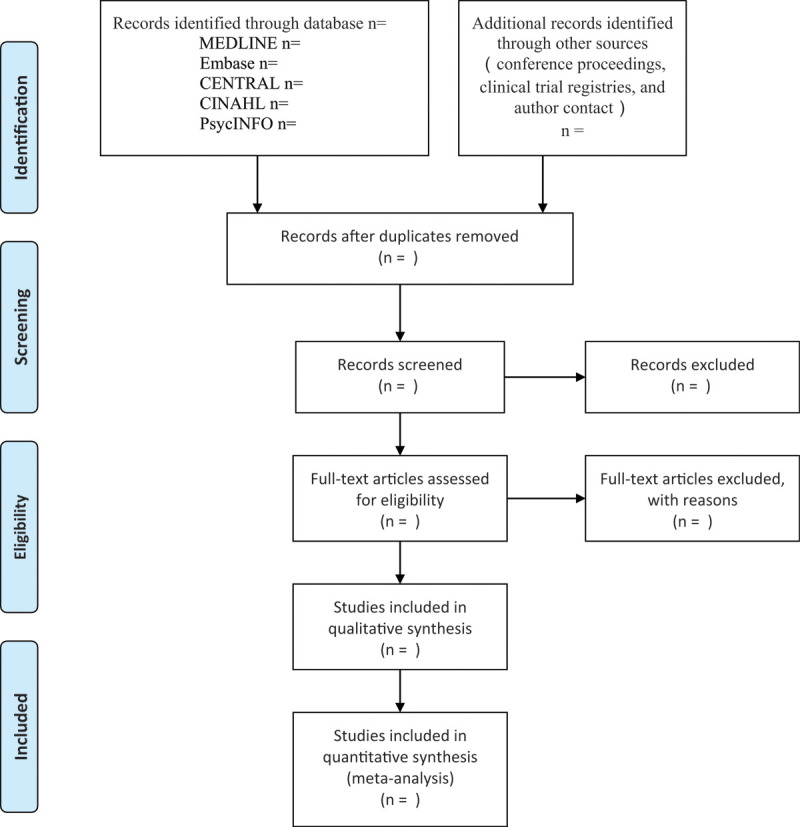
Study selection flow diagram.

Our analysis will only include RCTs of high quality in English which appraise the efficacy or safety of any pharmacological or non-pharmacological interventions treating patients with migraine. According to the eligibility criteria discussed above, the evaluation and screening of articles will be performed by 2 reviewers independently. And if there is any controversy after elaborate discussion, a third reviewer should then intervene to call the final determination. All the included RCTS will be organized into Endnote X9, a data management software. After deleting the duplicates, 2 reviewers will scan and screen the titles and abstracts of the left literature to decide which of them are worth being reviewed full text. Based on a rigorous and scientific review of full text, the finally included RCTs will be recognized in accord with the criteria. Whenever it is unclear if a study meets our criteria of incision, we will try our best to contact the author to ask for further information. Additionally, a calibration exercise will be applied since the selection start, which means each reviewer will be required to independently screen 10% of a random sample of articles at least to ensure appropriate inter-rater agreement (at least 80% agreement). The results of study selection flow diagram are shown in Fig. 2.

#### 2.3.2 Data extraction

Two of us will perform the data extraction independently and once the discrepancies come up, the final decision would be made by a third reviewer. Initially, the baseline characteristics of the included studies as potential effects modifiers will be abstracted roundly, and the information collected are as follow.

(1) Study characteristics: publication date, authorship, location of study, journal of publication, study sponsorships, etc.
(2) Patient characteristics: average age, proportion of female patients, occupations, etc.
(3) Intervention characteristics: intervention director, intervention protocol, medication dosing schedule, duration of medication, etc.

Secondly, the primary and secondary outcomes of safety and efficacy illustrated above will be extracted from included RCTs. The doses of drugs, methods of drug delivered, and schedules of drug administration will all be recognized from included RCTs.

#### 2.3.3 Node formation

This NMA is expected to identify various interventions for the treatment of migraine, while there is still no standardized taxonomy to classify them. For building a framework, we design a qualitative consensus-based categorization procedure, which consists of 4 steps. Identification, code, definition of all interventions from the previous systematic reviews and meta-analyses, independent classification of interventions into relevant fields (e.g., all interventions relating to psychological therapies would be sorted into this domain), settlement of the discrepancies in sorting the interventions through explicit discussion, connection of the representative figures (e.g., researchers, clinicians, allied health professionals, pharmacist, etc) to seek advice and reach the final consensus. Before all, the calibration exercise would be applied to make sure the reviewers could fulfill this process scientifically and rigorously. Each reviewer is required to identify and classify the interventions from 10% of a random sample of RCTs to achieve an appropriate inter-rater agreement (at least 80% agreement), which means a qualitative method of independent multiple coding of the interventions and a consensus approach integrating the stakeholders early in the analysis.

#### 2.3.4 Risk of bias assessment

The risk of bias of the selected RCTs will be accessed strictly following the criteria outlined in the Cochrane Handbook for Systematic Reviews of Interventions.^[32]^ By this Cochrane bias tools, 2 reviewers should independently evaluate the bias and quality of included RCTs from 6 aspects including random sequence generation (selection bias), allocation concealment (selection bias), blinding of participants and personnel (performance bias), blinding of outcome assessment (detection bias), incomplete outcome data (attrition bias), selective reporting (reporting bias), and other bias. Once there is any controversy, a third reviewer would give the final assessment and finally assign a level of risk of bias (high risk, unclear risk, low risk) for each item.

#### 2.3.5 Outcome measures

Our primary outcomes of efficacy are based on the difference of the number of responsive participants (dichotomous outcomes) between the interventions and comparators, which computed as odds ratio (OR) and credibility interval (CI). The same is some of our secondary outcomes of efficacy including headache frequency per month, headache severity, and headache duration, but the others are collected as the differences in scores of scales (continuous outcomes) thus being computed as mean difference (MD) and CI. And the outcomes of safety are all composed of the difference in the number of adverse events between the interventions and comparators, which are computed as risky ratio (RR) and CI. We will categorize the interventions by its generic name for pharmacological interventions (such as carbamazepine, amitriptyline) or the known modality for non-pharmacological interventions (such as cognitive behavioral therapy, transcranial magnetic stimulation). Also, if there are combined interventions, they would be recorded exactly as the combined name, for example, citalopram and recognizable psychotherapeutics.

### 2.4 Data synthesis and statistical analysis

#### 2.4.1 Baseline characteristics synthesis

The baseline characteristics data of included trials will be collected and descriptively summarized focusing on aspects of the study characteristics, patient characteristics, intervention and outcome measures, our assessment of the risk of bias, etc. If quantitative synthesis is not appropriate, we will then describe the results of the systematic review.

#### 2.4.2 Geometry of the network

Initially, we will conduct a geometry of the network of comparisons across trials to make sure that each included RCT would be connected in this NMA as well as exclude the unconnected ones. Each node in the network geometry represents a kind of intervention. Nodes will be linked by a line when the treatments are directly comparable with “head to head trails.” The size of the nodes corresponds to the number of participants receives that intervention and the width of the lines is proportional to the number of RCTs this comparison included.

#### 2.4.3 Pairwise meta-analysis

Then, we will perform traditional pairwise meta-analyses to anticipate the heterogeneity and publication bias among the RCTs before NMA. The characteristics of the included RCTs will be carefully examined to access the clinical and methodological heterogeneity. The heterogeneity will be assessed by *I*^2^ statistic and the publication bias judged through Begg^[33]^ and Egger^[34]^ funnel plots. And once the heterogeneity is anticipated, a random-effects model will be preferred to the fixed-effects model,^[35]^ and thus we will expect to utilize the random-effects model.^[35]^ All this process will be realized in Review Manager 5.3.3 (Cochrane Collaboration, Denmark) and the outcome measurements are the same as discussed above.

#### 2.4.4 Network meta-analysis

Next, the network meta-analysis is conducted within a Bayesian hierarchical model framework to obtain estimates for all the included valuable treatments for migraine. The random-effect model will be adopted because it is the most appropriate and advisable methodology in consideration of the between-study heterogeneities.^[36,37]^ In the NMA, we will run the Markov Chains Monte Carlo method with 4 chains, and the models will run with different arbitrarily chosen initial values and with non-informative priors. Each chain will have at least 200,000 iterations to ensure model convergence and at least the first 2500 simulations will be discarded as burn-in. The actual discarding of iterations and the number of thinning will refer to model convergence accessed through the Brooks–Gelman-Rubin plots method.^[38]^ All the process will be conducted using JAGS V.4.2.0, with “Gemtc,” “R2WinBUGS,” “rjags,” and “R2jags” packages in R V.3.6.0.

#### 2.4.5 Assessment of heterogeneity

Heterogeneity generally refers to the degree of disagreement among specific intervention effects and constitutes the majorly to the basis of inconsistency.^[39]^ The entire heterogeneity will be quantified with *Q* statistic and *I*^*2*^ index.

#### 2.4.6 Assessment of transitivity and similarity

We will cautiously assume the transitivity and similarity based on that the clinical and methodological characteristics described above are balanced on average across treatment comparisons.^[40]^ This assumption is set after reviewing all data of studies’ and participants’ characteristics and examining all potential efficacy modifiers such as age, timing of exposure, risk-of-bias, etc. And these effect modifiers will be judged and reported before the network meta-analysis is conducted. We will also carefully evaluate the treatment groups received comparative interventions (usual care or placebo) to make sure they are similar across pairwise comparisons.^[41]^

#### 2.4.7 Assessment of inconsistency

We will access the consistency of the whole network within the design-by-treatment interaction model.^[42]^ Once there is inconsistency observed in the network, we will then appraise the local inconsistency of each network loop using loop-specific method to generate an inconsistency factor with an associated 95% CI.^[43,44]^

#### 2.4.8 Sensitivity analysis

Sensitivity analyses would be processed incorporating only the RCTs at low risk of bias into the network estimates. And then we would assess the robustness of our results through a series of sensitivity analyses: the exclusion trials with a high risk of bias, the iterative removal of one study at a time, and the use of both fixed and random-effects models.

#### 2.4.9 Other analyses

We will perform additional analyses to enhance the scientificity and preciseness of this NMA. Network meta-regression would be realized using a random effects network to examine potential factors further. If we still finally tracked the inconsistency with no discrepancy to blame, subgroup analyses would be done on the potential factors identified through meta-regressions to explore possible sources of heterogeneity.

### 2.5 Assessment of quality of evidence

In our study, we adopt the widely acknowledged Grading of Recommendations, Assessment, Development and Evaluation (GRADE) system to access the quality of evidence on the efficacy and tolerability of comprehensive treatments for migraine. The features of GRADE system are a priority definition of outcomes and their relevance, as well as a distinction between the quality of evidence (also referred to as confidence in the estimate of intervention effect) and the strength of recommendations.^[45]^ Accordingly, we will evaluate the evidence based on 5 key aspects including methodology quality, directness of evidence, heterogeneity, precision of effect estimates, and risk of publication bias. Each evidence will be attributed to 1 of 4 levels—high, moderate, low, and very low.^[46]^

## 3 Discussion

This will be the first attempt to quantitatively synthesize the efficacy and tolerability of all available therapies for migraine, since all the previous reviews or meta-analyses focused on only one or several treatments ignoring the comprehensive interventions used in clinical. The NMA method can ensure us to fully utilize both the direct and indirect evidence as well as obtain the comparative estimates displayed in the derived hierarchies. Moreover, the NMA is designed to merely included RCTs with rigorous the incision/exclusion criteria, and we will also strictly follow the guidelines in PRISMA-NMA extension statement for reporting systematic review and NMA of health care interventions.

However, there would still be 2 anticipated challenges and maybe limitations in this systematic review and NMA. Firstly, the heterogeneity among the trials might hardly be eliminated totally, due to the differences in the dose, intensity, duration, delivery method of each intervention, etc. To face this challenge, the traditional pairwise meta-analyses will be conducted to anticipate the amount of heterogeneity with *I*^2^ statistic and the *Q* test. Once observed the heterogeneity, network meta-regression, and subgroup analyses will be performed to explore the potential effect modifiers. Moreover, comparison-adjusted funnel plots will help monitor the impact of such decision and any possible publication bias. Secondly, maybe some of the included interventions own little or unavailable data of clinical trials, and thus the conclusions of that would provide limited hints. To address this limitation, we will try to contact with the authors to gain the precise data, otherwise we would pay more attentions to interpreting the results avoiding exaggerating or ignoring the effects or tolerability.

## 4 Ethic and dissemination

This systematic review and NMA aims to synthesis quantitative and comparative conclusions on the efficacy and tolerability of all the potential interventions for treating migraine, utilizing all the available data of high-quality clinical trials. The most consolidated evidence would guideline the clinicians to make the best choice of interventions, from all the treatments no matter the pharmacological or the non-pharmacological. This study is expected to be completed until August 2020 and published in peer-reviewed journal.

## Author contributions

Boru Jin, Huayan Liu, and Lei Qiao conceived the study and drafted the manuscript. Boru Jin and Lei Qiao provided search strategies and professional advice. Boru Jin and Lei Qiao implemented a preliminary search. Boru Jin and Huayan Liu provided guidance on the NMA methodology. Lei Qiao and Huayan Liu provided expertise on treatments, outcomes and related knowledge on migraine. All authors read, critically reviewed, and approved the final manuscript as submitted.

## Abbreviations

Abbreviations: CENTRAL = Cochrane Central Register of Controlled Trials, CI = credibility interval, CINAHL = Cumulative Index to Nursing & Allied Health, GRADE = Grading of Recommendations, Assessment, Development and Evaluation, HIT-6 = headache impact test, MAAS = mindfulness attention awareness scale, MD = mean difference, MIDAS = migraine disability assessment, NMA = network meta-analysis, OR = odds ratio, PRISMA-P = Preferred Reporting Items for Systematic Review and Meta-Analysis Protocols, PROSPERO = International prospective register of systematic review, RCTs = randomized controlled trials, RR = risky ratio, SF-36 = 36-item short form survey.
